# Diversity of terrestrial mammal seed dispersers along a lowland Amazon forest regrowth gradient

**DOI:** 10.1371/journal.pone.0193752

**Published:** 2018-03-16

**Authors:** Alexander Arévalo-Sandi, Paulo Estefano D. Bobrowiec, Victor Juan Ulises Rodriguez Chuma, Darren Norris

**Affiliations:** 1 Programa de Pós-graduação em Ecologia, Instituto Nacional de Pesquisas da Amazônia (INPA), Av. André Araújo 2936, Petrópolis, Manaus, AM, Brazil; 2 Programa de Pós-Graduação em Biodiversidade Tropical, Universidade Federal do Amapá (UNIFAP), Rod. Juscelino Kubitscheck, Km 02, Macapá, AP, Brazil; 3 Facultad de Ciencias Forestales, Universidad Nacional de la Amazonía Peruana (UNAP), Pevas 5ta cdra, Iquitos, Perú; 4 Coordenação de Ciências Ambientais, Universidade Federal do Amapá (UNIFAP), Rod. Juscelino Kubitschek Km 02, Macapá, AP, Brazil; Sichuan University, CHINA

## Abstract

There is increasing interest in the restoration/regeneration of degraded tropical habitats yet the potential role of natural regenerators remains unclear. We test the hypothesis that the richness and functional diversity of terrestrial mammals differs between forest regrowth stages. We quantified the richness and functional diversity of eight terrestrial mammal seed-disperser species across a forest regrowth gradient in the eastern Brazilian Amazon. We installed camera-traps in 15 sites within small-holder properties with forest regrowth stage classified into three groups, with five sites each of: late second-regrowth forest, early second-regrowth forest and abandoned pasture. Species richness and functional dispersion from the regrowth sites were compared with 15 paired forest control sites. Multi model selection showed that regrowth class was more important for explaining patterns in richness and functional diversity than other variables from three non-mutually exclusive hypotheses: hunting (distance to house, distance to river, distance to town, small holder residence), land cover (% forest cover within 50 meters, 1 kilometer and 5 kilometers) and land use (regrowth class, time since last use). Differences in functional diversity were most strongly explained by a loss of body mass. We found that diversity in regrowth sites could be similar to control sites even in some early-second regrowth areas. This finding suggests that when surrounded by large intact forest areas the richness and functional diversity close to human small-holdings can return to pre-degradation values. Yet we also found a significant reduction in richness and functional diversity in more intensely degraded pasture sites. This reduction in richness and functional diversity may limit the potential for regeneration and increase costs for ecological regeneration and restoration actions around more intense regrowth areas.

## Introduction

Agriculture continues to drive forest loss and fragmentation across the Brazilian amazon [1, 2]. The loss and fragmentation of tropical forests causes drastic losses of species diversity and biotic interactions at local, regional and global scales [3, 4]. One option to revert this loss of diversity is the restoration of degraded forests and deforested landscapes [5]. However active restoration actions are often considered to be economically expensive, especially in developing nations. Faced with limited resources there is increasing interest in integrating natural regeneration within restoration actions [5].

Plant-animal interactions are fundamental for tropical forest biodiversity [6] and the recovery of faunal diversity and associated ecosystem services (e.g. seed dispersal) are fundamental for natural regeneration processes in degraded forests [5, 7]. Mammals play an important role in the recovery processes of altered areas, because of their capacity for dispersal and predation of seeds and seedlings of many plant species [8]. Medium and large-bodied mammals (weight> 1 kg) can disperse a large numbers of seeds over long distances [8, 9]. For example, lowland tapirs can travel over 4 kilometers per day [10] and disperse seeds of more than 70 species with different types and sizes of seeds in a single location [11]. The absence of large mammals may release some plant species from the herbivory and increase their dominance, which consequently decreases biodiversity in forests [12, 13].

Traditional analyzes of biological diversity such as number of species, abundance and composition consider that the species of an assembly are phenotypically similar and do not necessarily reflect the intrinsic ecological and evolutionary attributes of the species [14–16]. Functional traits are measures of diversity that incorporate phenotypic information from species that directly affect the individual's performance in the environment [16, 17]. Functional traits allow evaluation of how morphological, physiological and/or behavioral characteristics respond to changes along an environmental gradient [18]. Degraded areas at different stages of regeneration filter which species with specific functional traits persist in secondary vegetation [19, 20]. These traits allow species to meet their ecological requirements in degraded forest landscapes.

The responses of medium and large-bodied terrestrial mammals to habitat loss and fragmentation have been extensively evaluated for taxonomic diversity, such as species number, abundance and composition [21–23]. However, it is not yet known how the functional diversity of medium and large-bodied mammals respond to the effects of forest degradation and which functional traits are sensitive to changes in the quality of degraded habitats. In this study we analyzed the changes in functional and taxonomic diversity of medium and large-bodied terrestrial mammals important for natural regeneration, especially seed dispersal along a degradation gradient (intact forest, late second-regrowth forest, early second-regrowth forest, abandoned pasture) in *terra-firme* forest in the eastern Brazilian Amazon. Our hypothesis is that areas that have suffered degradation in recent years (early secondary-regrowth forest) or with greater intensity (abandoned pasture) present greater loss of taxonomic and functional diversity compared to areas of late secondary-regrowth and intact forest. We predict that functional diversity and number of species will be reduced or lost in areas that have been recently or more intensely degraded.

## Methods

### Ethics statement

Data collection used non-invasive, remotely activated camera traps and did not involve direct contact or interaction with animals, thus no ethical approval was required. Fieldwork was conducted under research permit number IBAMA/SISBIO 40355–1 and 47859–2 to DN, issued by the Instituto Chico Mendes de ConservacËão da Biodiversidade (ICMBio). Interviews with local residents were approved by IBAMA/SISBIO (permits 45034–1, 45034–2, 45034–3) and the Ethics Committee in Research from the Federal University of Amapá (UNIFAP) (CAAE 42064815.5.0000.0003, Permit number 1.013.843).

### Study area

This study was conducted in private properties surrounding the Amapá National Forest (Floresta Nacional Amapá —hereafter ANF). ANF is a sustainable-use (IUCN protected area category IV) protected area of approximately 460,000 ha [24], located on the pre-Cambrian Guianan shield craton at the base of the Tumucumaque Uplands, in the northeast Brazilian Amazon (0°55’29”N, 51°35’45”W, S1 Text). The regional phytophysiognomies consist of evergreen tropical rainforest vegetation [25], predominantly never flooded “*terra-firme*” forest, with some areas of flooded forest, bamboo and rocky outcrops [24]. The regional climate is classified by Köppen-Geiger as Am (Equatorial monsoon, [26]) with annual rainfall ranging from 2,200 mm to 2,500 mm during the last five years (2012–2016, [27]). During the months with highest precipitation levels (February, March and April), rainfall may reach 500 mm/month. The dry season (September to November) is characterized by precipitation below 150 mm/month ([27], S1 Fig).

The ANF is part of a large (> 4 million hectares) connected group of protected areas (S1 Text, [24]) that maintain both continuous undisturbed forests and the complete regional community of medium-sized and large-bodied vertebrates [24, 28–30]. The presence of medium-sized and large-bodied vertebrates that are often preferred hunter targets [31] can be largely explained by the relatively low human population densities in the study area. The most recent census provides an estimate of 3.8 habitants/km^2^ in the area (Porto Grande municipality, [32]). Although population density across Brazilian Amazonia ranges widely (from <1 to >1000 inhabitants per km^2^), the population density in our study area is representative of the most frequently encountered human population density across Brazilian Amazonia ([32], see supplemental material S1 in Norris and Michalski [33]).

### Sampling design

Data were sampled during the wet-dry season transition (May to September 2016), with total monthly rainfall ranging from 80–171 mm (S2 Fig). This period corresponds to the peak/end of fruiting of lowland Amazon forests in the study region [34–36], and as such was chosen to obtain the best possible sample of terrestrial frugivores expected to be active during periods of fruit and seed availability [37–39]. Data collection was conducted in 15 *terra-firme* sites located in private small-holder properties that were selected on the basis of differences in land-use histories and forest succession/regeneration stage (S1 Text). All sites were close (110 – 554m) to 100–200 m wide rivers that are navigable by motorized boats, but due to riverbank formation the sites are never flooded. These 15 sites were grouped into three degradation classes based on the land-use history: late second-regrowth forest (N = 5, most recent human disturbance between 20 and 25 years), early second-regrowth (N = 5, most recent human disturbance between 1 and 5 years), and pasture (N = 5, recently cleared and abandoned pasture areas dominated by grasses/herbs but that had never been used to raise livestock, with the most recent disturbance between 1 and 17 years). Each of the 15 regrowth camera-trap sites was paired with a camera-trap in a nearby (60 to 150 m) control site i.e. 20–30 m tall *terra-firme* forest site without a history of mechanized timber extraction. To reduce the possible confounding influence of edge effects that are known to strongly influence the distribution of the study species in neotropical forests [40], all regrowth and control sites were established at a standardized distance (approximately 30 m) from the nearest control-regrowth habitat edge.

### Mammal survey

To sample mammal seed dispersers, we installed camera traps equipped with infrared triggers (Bushnell Trophy Cam, 8MP, Overland Park, KS, USA) in each of the 30 sites. Cameras were placed at 14 sites for 30 consecutive days (June-July 2016) then immediately transferred to the remaining 16 sites (July-August 2016). Each of the three regrowth classes was sampled in both temporal blocks and as the sampling was conducted over a relatively short period (three months) we assume that this division does not introduce any systematic bias.

The locations of camera trap installation were selected in a two stage process. Firstly we conducted interviews with 36 local landowners along 110 km of river to identify properties with areas that had suitable land-use histories. Once the properties were selected, the areas with distinct regrowth ages on each property were mapped using a handheld GPS with the aid of the local landowners who knew the history of land use. We then overlaid the regrowth polygons within a GIS to determine the potential location of installation points based on standardized criteria (detailed in “Sampling design” and including distance from river, distance from edge, proximity to control sites). We then returned to the sites to install cameras. New access paths (< 1 m wide) were cut with a hand held machete to access installation locations of camera traps. All cameras were unbaited, installed 30–40 cm above the ground and programmed to film for 40 seconds post-activation, with 10 second intervals between videos. An area (approximately 7 x 7 m) in front of the cameras was cleared of green foliage and herbs to prevent sunlight reflections damaging image quality and the movement of vegetation falsely triggering cameras. Cameras functioned continuously (24 hours a day) during the 30-day sample period. In some of the pasture sites, we had to exclude a number of collection days due to memory cards becoming full and batteries finishing. The species recorded by the camera traps were identified with the aid of field guides [41–43]. Scientific names follow Paglia, da Fonseca (42).

### Functional diversity assessment

We considered as ‘functional’ a morphological or behavioral trait that can be relevant to ecosystem functioning or filtered by environmental gradients (natural or human-induced) [44]. We selected four traits (trophic guild, group living, body mass, and home range, Table 1) that influence/characterize species interactions with the tropical flora including seed dispersal, herbivory, seed predation, and trampling [8, 13]. Trophic guild (categorical, with 3 classes: scatter-hoarder, folivore-frugivore and omnivore-frugivore [37, 42, 45]), group living (binomial, yes or no [46]), and body mass (log transformed for analyses, [42]) reflect the type, distance and amount of fruits and seeds that species consume. Home range (categorical, four classes: 1 = 0–10 ha; 2 = 11–50 ha; 3 = 51–100 ha; 4 = >100 ha, [46, 47]) strongly influences the spatial distribution and extent to which a species travels in search of food and disperses seeds [8, 13].

**Table 1:** Functional traits and number of independent videos (detections) and relative abundance (RA) of eight mammal species along a forest regrowth gradient in the eastern Amazon.

		Functional traits				FG^j^	Presence^k^		Detections^l^		RA^m^	
Order/Species		GL^a^	BM^b^	HR^c^	TG^f^		Con.	Regr.	Con.	Regr.	Con.	Regr.
Rodentia												
	*Cuniculus paca*	No	9.3	1^d^	FF^g^^,^^h^	2	7	3	12	14	0.27	0.37
	*Dasyprocta leporina*	No	5.5	1^d^	SH^g^^,^^h^^,^^i^	1	**14**	**9**	**88**	**28**	**2.00**	**0.74**
	*Myoprocta acouchy*	No	1	1^d^	SH^g^^,^^h^	1	6	4	**17**	**5**	0.38	0.13
Artiodactyla												
	*Mazama americana*	No	36	3^d^^,e^	FF^g^^,^^i^	2	4	6	9	11	0.20	0.29
	*Mazama nemorivaga*	No	20	2^d^	FF^g^^,^^i^	2	2	2	5	2	0.11	0.05
	*Pecari tajacu*	Yes	26	4^d^	OF^g^^,^^i^	3	**10**	**3**	**27**	**5**	**0.60**	**0.13**
	*Tayassu pecari*	Yes	35	4^d^	OF^g^^,^^i^	3	1	1	1	2	0.02	0.05
Perissodactyla												
	*Tapirus terrestris*	No	260	4^d^	FF^g^^,^^i^	4	3	1	3	3	0.07	0.08
	Totals						**15**	**10**	**162**	**70**	**3.60**	**1.86**

^a^ Group living, from Jones, Bielby (46).

^b^ Mean adult body mass, in kg (log transformed for analyses). From Paglia, da Fonseca (42).

^c^ Home range class. 1 = 0–10 ha; 2 = 11–50 ha; 3 = 51–100 ha; 4> = 100 ha. From

^d^Jones, Bielby (46)

^e^Maffei and Taber (47).

^f^ Trophic guild. FF = Folivore-frugivore, SH = Scatter-hoarder, OF = Omnivore-frugivore, from

^g^Paglia, da Fonseca (42)

^h^Arita, Robinson (45)

^i^Bodmer (37).

^j^ Functional group. Species with the same number belong to the same functional group as defined from the trait distance matrix using K-means clustering.

^k^ Site presence, defined as the number of camera-traps with at least one video. Bold shows significant (*P* < 0.1) differences in the proportion of camera-traps with at least one video between control (“Con.”) and regrowth (“Regr.”) areas.

^l^ Total detections with independent videos. Bold shows significant (*P* < 0.1) differences in mean RA between control and regrowth areas (GLM, family = poisson, link = log).

^m^ Relative abundance (RA) expressed as the number of independent videos per 10 camera-trap days. Bold shows significant (*P* < 0.1) differences in mean RA between control and regrowth areas (one-way ANOVA with White-corrected covariance matrix).

#### Data analysis

All analysis were conducted using the R language and environment for statistical computing [48], with base functions and functions available in the following packages: *vegan* [49], *FD* [50], *ggplot2* [51] and *MuMIn* [52]. To estimate the relative abundance of mammals, we considered only independent videos, with over 30 min intervals when the same species was recorded during the same day on the same camera [28, 53]. The relative abundance of each species was expressed as the number of independent videos per 10 trap-days [21, 28, 53]. To assess whether the sampling effort was sufficient to record the eight species in the regrowth and control sites and the three degradation classes, we compared cumulative species curves (specaccum function [49]).

We calculated a richness and functional diversity (FD) value for each of the 30 sites. Richness was calculated as the observed number of species (hereafter “species richness”) at each site. Although there are myriad taxonomic diversity metrics, we chose species richness as it is both widely used and clearly interpretable [54, 55] and with relatively few (eight) species and 30 sites there were strong correlations between species richness values and alternative diversity metrics such as Shannon and Simpson diversity (Spearman rho > 0.89). We used Functional Dispersion (FDis) [56] as an index of functional diversity. We used FDis because it is not strongly influenced by outliers, accounts for relative abundances, is unaffected by species richness and can be calculated from any distance/dissimilarity measure [50, 56]. Functional Dispersion was estimated with the dbFD function [50] using the function default settings.

We used analysis of variance (ANOVA) to compare the species richness and functional dispersion between regrowth classes. To ensure assumptions of ANOVA (i.e. homogeneity of variance) were met, the ANOVAs were run based on the White-corrected covariance matrix [57, 58]. To examine patterns in richness, functional diversity and the pair-wise differences between regrowth and control sites we used linear regressions. Preliminary examination of model residuals showed that Generalized Linear Models with a Tweedie error distribution did not improve model fits compared with linear models, we therefore ran all analysis using linear regression. The regression approach was preferred to alternatives such as occupancy models because the number of videos (i.e., potential of recaptures) and naïve occupancy (proportion of cameras with records) was low for most species. We used an information theoretic model averaging framework [59] to examine the support for three models representing three non-mutually exclusive hypotheses–hunting pressure, forest cover and land use. Each model included the following explanatory variables (see S1 Table for variable description and justification for inclusion): hunting pressure (distance to house, distance to river, distance to town, small holder residence), land cover (% forest cover within 50 meters, 1 kilometer and 5 kilometers) and land use (regrowth class, time since last use). We evaluated models based on their information content, as measured by AICc–Akaike Information Criterion corrected for small sample sizes. With a strong a priori justification for inclusion, we retained all variables (minimally-correlated within each model, pairwise Spearman’s correlation r < 0.62), and all possible candidate models; therefore, all variables were on equal footing to calculate their relative importance as measured by the variables’ Akaike weights (Burnham & Anderson 2002 pp. 75–77, 167–172), which is a scaled measure of the likelihood ratio that ranges between 0 (least important) and 1 (most important). To calculate average values for slopes, we used a reduced subset of models for a 95% confidence set. This confidence set was obtained by summing the Akaike weights of the set of all candidate models ordered by Akaike weight from largest to smallest until a sum of 0.95 was obtained (Burnham & Anderson 2002 pp. 169, 176–177). To reduce model selection bias, model averaging was carried out using the full set of confidence models, i.e. when a predictor was not present in the model its value was set to 0 (Burnham and Anderson 2002, pp. 152). None of the unexplained variation (model residuals) was related to the geographic distance among camera-traps (S2 Fig), so we did not need to control for spatial dependence.

To assess which of the terrestrial mammal functional traits are sensitive to changes in regrowth stage, we compared an index of change (% difference in relation to control sites) between species in the different regrowth classes. Abandoned pasture was not included because no species was recorded in this class. We used the value of the record change as a response variable and the functional traits as the explanatory variables. We ran six GLMs, three with single traits (body mass, trophic guild, and home range) and three with two traits. The trait social group size was not included because it did not present enough variation to run the GLMs. We selected the best GLMs based on AICc using the R package MuMIn [52]. We considered models with strong support to be those with ΔAIC values less than two and high (i.e. close to 1) Akaike weight values [59].

## Results

### Sampling effort and mammal diversity

From a sampling effort of 827 camera-trap days (450 and 377 camera-trap days, control and regrowth sites respectively), we obtained an overall capture rate of 0.28 videos per trap-day (232 independent videos/ 827 trap-days, Table 1). Overall, there were less detections (independent videos) in regrowth compared with control areas (162 and 70 independent videos, control and regrowth areas respectively, Table 1). The number of detections also declined with increasing degradation intensity (44, 26, 0 independent videos, late second-regrowth, early second-regrowth, pasture respectively).

**Fig 1:**
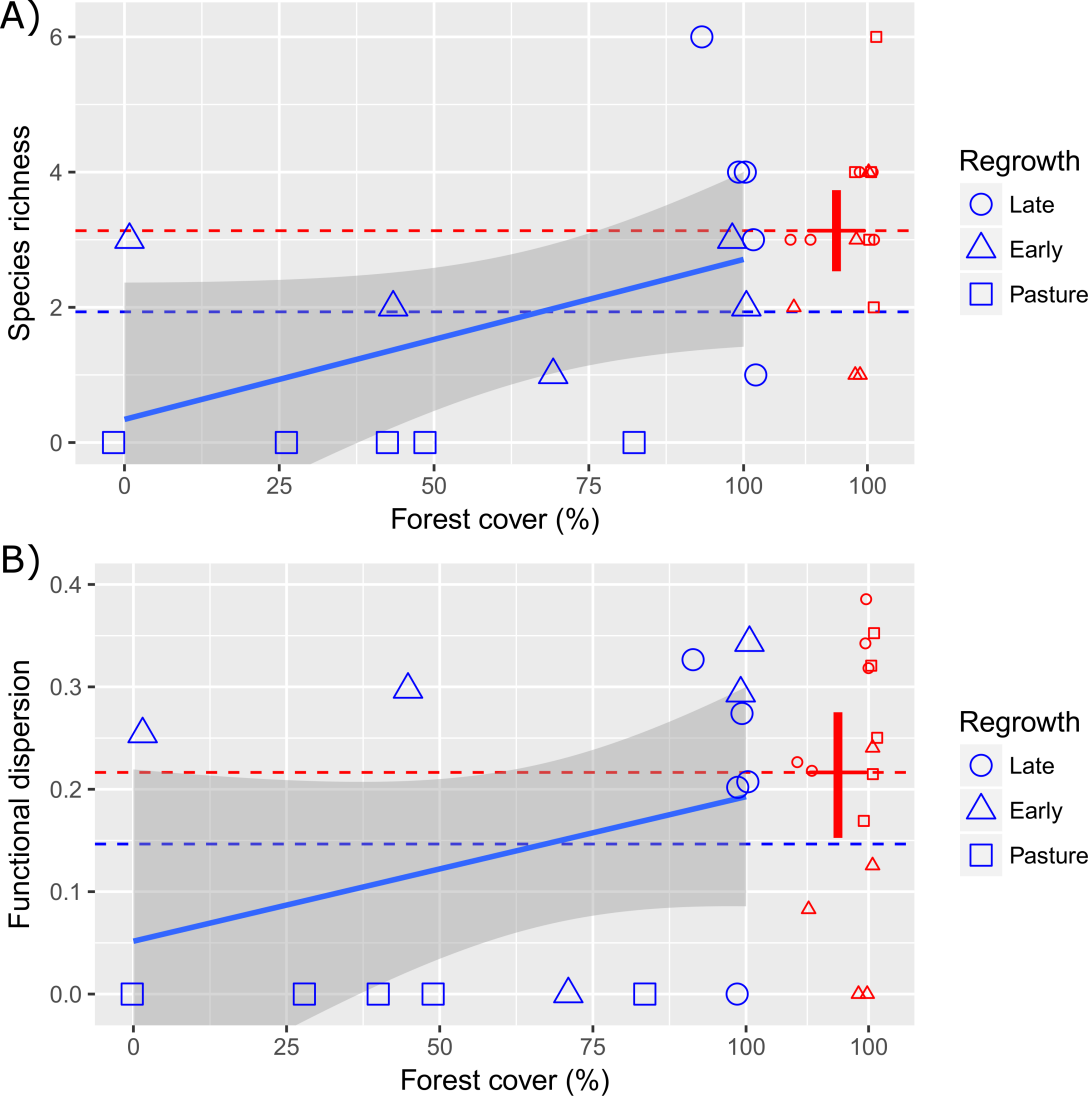
Mammal diversity in Amazon regrowth forests. Patterns of terrestrial mammal species richness and functional dispersion surrounding lowland Amazon small-holdings. Showing the percentage forest cover within a 50 meter radius of 30 camera-trap stations used to record the presence of eight mid to large-bodied terrestrial mammal seed dispersers. Differences in richness and functional dispersion obtained from camera-trap videos taken in three regrowth classes (blue symbols, N = 5 sites each) compared with paired control sites (red symbols). Control sites are moved to the right along the x axis for clarity. Solid red crosses represent mean (horizontal bar) and 95% confidence limits (vertical line) of the 15 control site values estimated via nonparametric bootstrap. Horizontal dashed lines show mean values (regrowth and controls, red and blue respectively). Solid blue line shows the linear trend and 95% confidence intervals (grey shading).

We recorded between zero and six mammal species at each site (Fig 1). The species accumulation curves tended to stabilize (i.e. approached an asymptote), suggesting that sampling effort was sufficient to detect the eight mammal species when all (N = 30), control (N = 15) and regrowth (N = 15) sites were considered (S3 Fig). All eight species were recorded in late second-regrowth sites, where the accumulation curve also approached an asymptote (S4 Fig). In contrast a total of five (63%) species (*Dasyprocta leporina*, *Myoprocta acouchy*, *Mazama americana*, *M*. *nemorivaga* and *Pecari tajacu*) were recorded in the early second-regrowth sites (S4 Fig). None of the eight species were recorded on the abandoned pasture.

### Richness and functional diversity

Forest cover around the camera trap locations significantly explained variation in the richness and functional dispersion across the 30 sites (Fig 1, linear regression species richness R^2^_adj_ = 0.23, P-value F_1, 28_ = 0.004 and linear regression functional dispersion R^2^_adj_ = 0.11, P-value F_1, 28_ = 0.04). Yet, there remained substantial variation in the number of species and functional dispersion that could not be explained by the percentage forest cover surrounding the camera traps at the 15 regrowth sites (linear regression species richness R^2^_adj_ = 0.16, P-value F_1, 13_ = 0.076 and linear regression functional dispersion R^2^_adj_ = 0.06, P-value F_1, 13_ = 0.19). For example the number of species encountered in control and late regrowth sites (all with forest cover >88%) varied between 1 and 6 (Fig 1). The regrowth stage affected the species encountered independent of forest cover, with the number of species recorded declining from regrowth (late to early) and pasture when the different sites had similar forest cover percentages (Fig 1).

**Fig 2:**
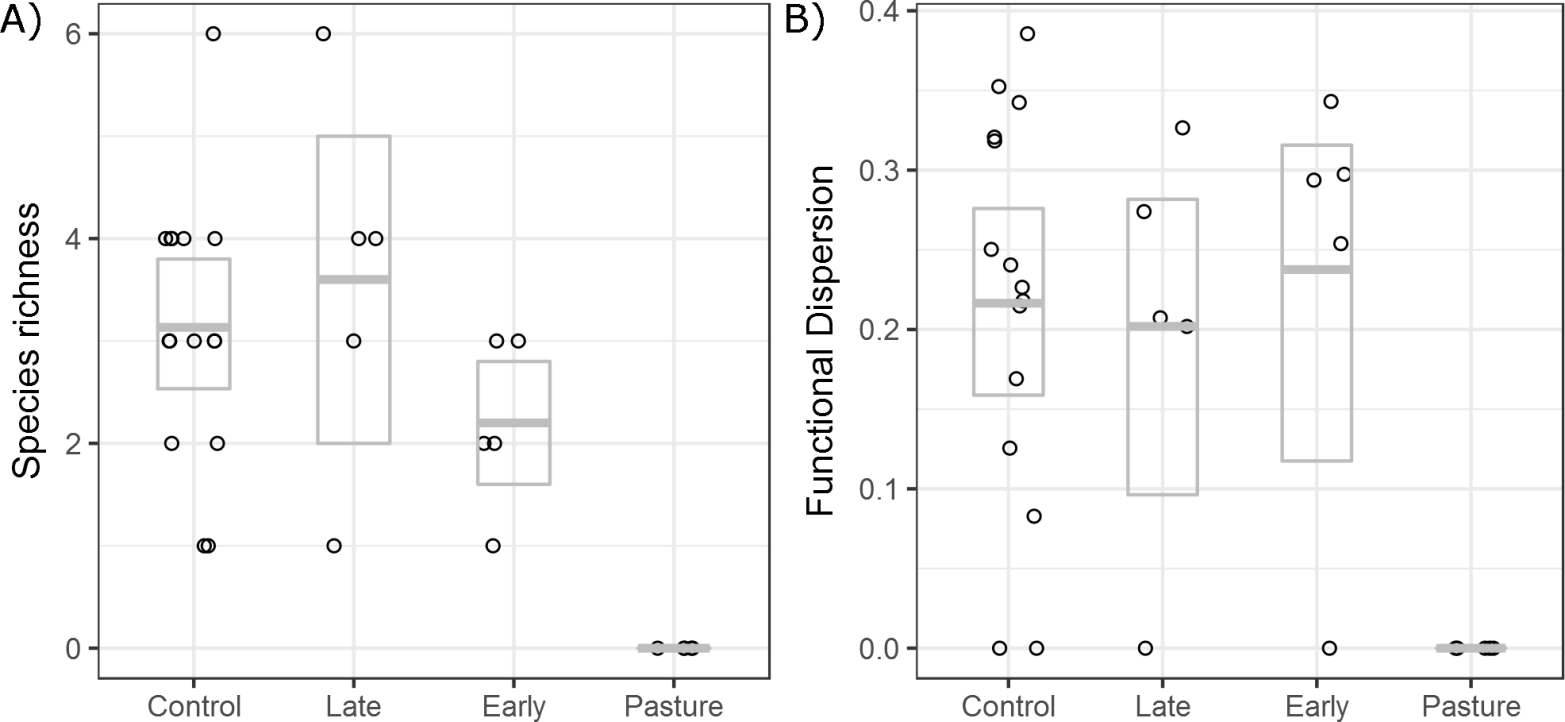
Mammal species richness and functional dispersion. Comparison of species richness and functional dispersion along a forest regrowth gradient in the eastern Amazon. Boxplots show means and 95% confidence limits estimated via nonparametric bootstrap.

Forest cover and hunting pressure were only weakly supported as informative in explaining the variation in species richness and functional dispersion (Table 2). Land use (regrowth stage and age since last use) was the most strongly supported model, explaining more than 50% of the variation in species richness and 30% of the variation in functional dispersion (Table 2). On average the number of species and functional diversity was reduced in regrowth areas compared with controls (Fig 1), but the reduction depended on regrowth stage (Fig 1, Fig 2, Table 2). There was a clear loss in species richness and functional dispersion in pasture areas, however several early and late second-regrowth sites showed high species and functional dispersion values (Fig 2). Richness declined with increasing degradation intensity, with a reduction in mean species richness in both early second-regrowth and pasture sites (Fig 2, Table 2). In contrast, both late and early second-regrowth classes retained similar mean functional diversity values to those found in control sites (Fig 2, Table 2).

**Table 2:** Model weights and parameter (slope) estimates from information-theoretic analysis of (a) species richness and (b) functional dispersion.

a) Species richness															
	Hunting			Forest cover			Land use			Global			Final		
* *	*B*	*CI* ^a^	*∑ w*_*İ*_ ^b^	*B*	*CI* ^a^	*∑ w*_*İ*_ ^b^	*B*	*CI* ^a^	*∑ w*_*İ*_ ^b^	*B*	*CI* ^a^	*∑ w*_*İ*_ ^b^	*B*	*CI* ^a^	*∑ w*_*İ*_ ^b^
(Intercept)	1.7	-0.1–3.6		2.5	2.0–3.1		7.0	2.9–11.1		7.3	2.7–11.8		6.9	2.7–11.0	
Dist. river	0.6	-0.2–1.4	0.54							0.2	-0.4–0.8	0.15			
Dist. town	0.4	-0.2–1.1	0.39												
Dist. house	0.0	-0.9–0.9	0.18												
Presence ^c^			0.90									0.46			0.51
Perm.	0.3	-1.8–2.4								-0.8	-2.2–0.7		-0.9	-2.1–0.4	
Semi-perm	**2.0**	**-0.2–4.1**								0.4	-1.3–2.1		0.3	-1.2–1.7	
Cover 50m				**0.8**	**0.3–1.4**	1.00				0.2	-0.6–1.0	0.07			
Cover 1km				**0.9**	**-0.1–1.8**	0.61				0.1	-0.5–0.7	0.04			
Cover 5km				-0.5	-1.4–0.5	0.29									
Regrowth ^d^									1.00			1.00			1.00
Late							**-6.8**	**-14.4–0.9**		**-7.2**	**-15.2–0.8**		**-6.1**	**-13.3–1.1**	
Early							**-9.2**	**-17.9–-0.5**		**-9.3**	**-18.3–-0.2**		**-8.1**	**-16.4–0.1**	
Pasture							**-10.9**	**-19.1–-2.7**		**-10.1**	**-18.5–-1.7**		**-9.4**	**-17.3–-1.6**	
Last use (Years)							**-4.0**	**-8.1–0.2**	0.65	**-4.0**	**-8.4–0.4**	0.65	**-3.4**	**-7.3–0.6**	0.57
R^2^ / adj. R^2^	**.39 / .26**			**.36 / .29**			.**59 / .52**			**.69 / .55**			**.67 / .59**		
AICc	120.1			114.9			104.8			117.4			105.3		
b) Functional dispersion															
	Hunting			Forest cover			Land use			Global			Final		
	*B*	*CI* ^a^	*∑ w*_*İ*_ ^b^	*B*	*CI* ^a^	*∑ w*_*İ*_ ^b^	*B*	*CI* ^a^	*∑ w*_*İ*_ ^b^	*B*	*CI* ^a^	*∑ w*_*İ*_ ^b^	*B*	*CI* ^a^	*∑ w*_*İ*_ ^b^
(Intercept)	0.1	-0.1–0.3		**0.2**	**0.1–0.2**		**0.4**	**-0.0–0.8**		**0.5**	**0.1–0.9**		**0.2**	**0.2–0.3**	
Dist. river	0.0	-0.0–0.1	0.42							0.0	-0.0–0.1	0.16			
Dist. town	**0.1**	**-0.0–0.1**	0.93							**0.1**	**0.0–0.1**	0.57	0.0	-0.0–0.1	0.50
Dist. house	0.0	-0.1–0.1	0.18												
Presence ^c^			0.14												
Perm.	0.1	-0.1–0.2													
Semi-perm	0.1	-0.1–0.3													
Cover 50m				**0.0**	**-0.0–0.1**	0.76				0.0	-0.1–0.1	0.23			
Cover 1km				0.0	-0.0–0.1	0.23				-0.0	-0.1–0.0	0.08			
Cover 5km				-0.0	-0.1–0.1	0.12									
Regrowth ^d^									1.00			0.71			
Late							-0.3	-1.1–0.4		-0.6	-1.4–0.2		-0.0	-0.1–0.1	0.86
Early							-0.4	-1.2–0.5		-0.6	-1.6–0.3		0.0	-0.1–0.1	
Pasture							-0.6	-1.4–0.2		**-0.8**	**-1.6–0.1**		**-0.2**	**-0.3–-0.1**	
Last use (Years)							-0.2	-0.6–0.2	.26	-0.3	-0.8–0.1	0.18			
R^2^ / adj. R^2^	.32 / .18			.17 / .08			.**39 / .29**			**.50 / .31**			**.42 / .33**		
AICc	-27.47			-28.32			-34.26			-24.34			-35.73		

Bold font shows significant variable slope estimates (P value of t statistic < 0.1) and significant model explanatory power (P value of model F statistic < 0.05)

^a^ Confidence interval of variable slope estimate.

^b^ The sum of Akaike weights (*w*_*İ*_) for all models with a given variable from a confidence sub-set of models with difference between model AICc values ≤ 4.

^c^ Presence shows difference responses from areas with permanent and semi-permanent human presence compared with abandoned sites.

^d^ Regrowth class shows slopes of difference responses from late second-regrowth, early second-regrowth and pasture sites contrasted with control forest sites.

**Fig 3:**
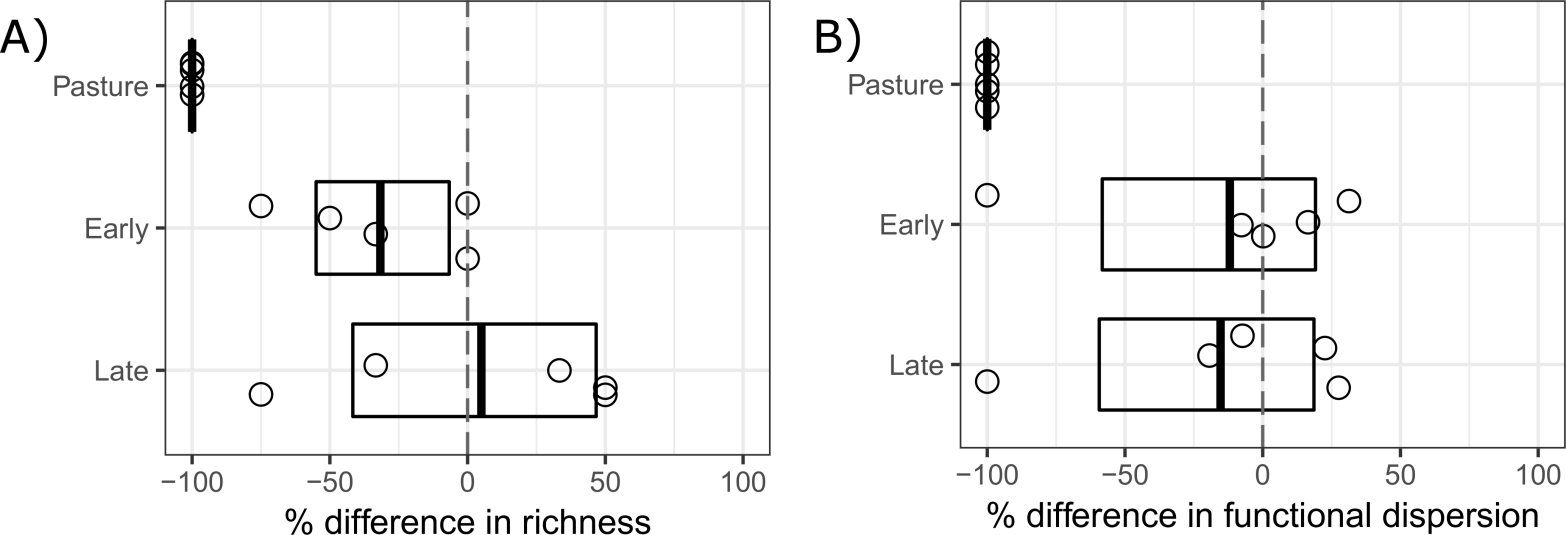
Differences in species richness and functional dispersion. Differences in species richness and functional dispersion of eight terrestrial mammal seed dispersers along a regrowth gradient in the eastern Amazon. Pair-wise percentage differences calculated using control sites as reference. Lower and higher species number and functional dispersion are represented by negative (−) and positive (+) values respectively. Boxplots show means and 95% confidence limits estimated via nonparametric bootstrap.

Species richness differed markedly between regrowth areas and the paired controls (Fig 3, Table 3, one-way ANOVA with White-corrected covariance matrix, F_2, 12_ = 15.8, P < 0.001). There was a significant reduction (95% CI did not overlap 0) in the number of species found in pasture and early secondary-regrowth areas (Fig 3). In contrast only pasture areas showed a significant reduction in functional dispersion (Fig 3) and there was no significant difference between regrowth class (Fig 3, Table 3, one-way ANOVA with White-corrected covariance matrix, F_2, 12_ = 0.99, P = 0.40). There was little support for any of the other models or covariates examined (Table 3). The late secondary-regrowth sites lost on average 15.3% in functional diversity but gained 5% in species richness compared with control areas (Fig 3). In contrast, the younger (early second-regrowth) sites suffered reductions in both mean functional and taxonomic diversity (-31.7% and -12%, species richness and functional dispersion respectively, Fig 3). With zero detections, pasture sites lost 100% of richness and functional diversity (Fig 3).

**Table 3:** Model weights and parameter (slope) estimates from information-theoretic analysis of percentage differences in (a) species richness and (b) functional dispersion.

a) Species richness															
	Hunting			Forest cover			Land use			Global			Final		
* *	*B*	*CI* ^a^	*∑ w*_*İ*_ ^b^	*B*	*CI* ^a^	*∑ w*_*İ*_ ^b^	*B*	*CI* ^a^	*∑ w*_*İ*_ ^b^	*B*	*CI* ^a^	*∑ w*_*İ*_ ^b^	*B*	*CI* ^a^	*∑ w*_*İ*_ ^b^
(Intercept)	-78.2	-166.1–9.7		-42.2	-70.1–-14.3		-34.1	-77.1–8.8		-37.4	-87.5–12.6		-36.9	-71.8–-2.0	
Dist. river	18.2	-23.8–60.2	0.11												
Dist. town	28.8	-7.2–64.9	1.00							18.1	-15.5–51.6	0.50	19.1	-5.2–43.3	0.46
Dist. house	-8.5	-51.5–34.5	0.13												
Presence ^c^			0.00												
Perm.	19.2	-87.2–125.6													
Semi-perm	67.5	-33.2–168.2													
Cover 50m diff				**25.4**	**-3.5–54.3**	0.51				-10.3	-48.9–28.2	0.09			
Cover 1km				**46.7**	**-4.8–98.2**	0.21									
Cover 5 km		.		-39.1	-90.6–12.5	0.28									
Regrowth ^d^									0.98			0.79			0.86
Early							41.7	-24.9–108.2		48.1	-26.4–122.6		33.4	-15.2–82.0	
Pasture							**-65.9**	**-123.4–-8.4**		-62.5	-154.8–29.7		**-49.2**	**-103.4–5.0**	
Last use (Years)							-3.7	-31.8–24.4	0.10						
R^2^ / adj. R^2^	.60 / .38			.42 / .26			**.63 / .53**			.70 / .59			**.71 / .63**		
AICc	179.0			171.4			164.6			166.2			161.1		
b) Functional dispersion															
	Hunting			Forest cover			Land use			Global			Final		
	*B*	*CI* ^a^	*∑ w*_*İ*_ ^b^	*B*	*CI* ^a^	*∑ w*_*İ*_ ^b^	*B*	*CI* ^a^	*∑ w*_*İ*_ ^b^	*B*	*CI* ^a^	*∑ w*_*İ*_ ^b^	*B*	*CI* ^a^	*∑ w*_*İ*_ ^b^
(Intercept)	-68.4	-186.6–49.7		-33.6	-68.5–1.3		-15.8	-71.3–39.8		-15.9	-98.8–67.0		-11.1	-61.2–34.9	
Dist. river	30.3	-29.2–89.7	0.09												
Dist. town	15.8	-34.7–66.3	0.30							5.4	-51.4–62.1	0.22			
Dist. house	-8.3	-71.4–54.8	0.10												
Presence ^c^			0.00												
Perm.	10.3	-135.0–155.6													
Semi-perm	67.0	-72.7–206.7													
Cover 50m diff				20.3	-16.6–57.2	0.19				-6.9	-84.1–70.3	0.14			
Cover 1km				42.0	-22.7–106.8	0.17									
Cover 5 km				-32.2	-97.4–32.9	0.10									
Regrowth ^d^									0.45			0.34			
Early							4.1	-82.1–90.2		0.7	-130.4–131.8		-5.2	-73.2–62.8	
Pasture							**-83.8**	**-168.7–1.0**		**-77.5**	**-255.6–100.6**		**-80.0**	**-163.0–3.0**	
Last use (Years)							-5.6	-45.0–33.7	0.09						
R^2^ / adj. R^2^	.41 / -.01			.27 / .03			.46 / .27			.49 / .24			.**45 / .34**		
AICc	170.3			155.2			151.4			158.0			141.0		

Bold font shows significant variable slope estimates (P value of variable t statistic < 0.1) and significant model explanatory power (P value of model F statistic < 0.05)

^a^ Confidence interval of slope estimate.

^b^ The sum of Akaike weights (*w*_*İ*_) for all models with a given variable. Weights obtained from confidence sub-set of models with difference between model AICc values ≤ 4.

^c^ Presence shows difference responses from areas with permanent and semi-permanent human presence compared with abandoned sites.

^d^ Regrowth class shows slopes of difference responses from early second-regrowth and pasture sites contrasted with control forest sites.

### Trait correlates of degradation gradient sensitivity

**Fig 4:**
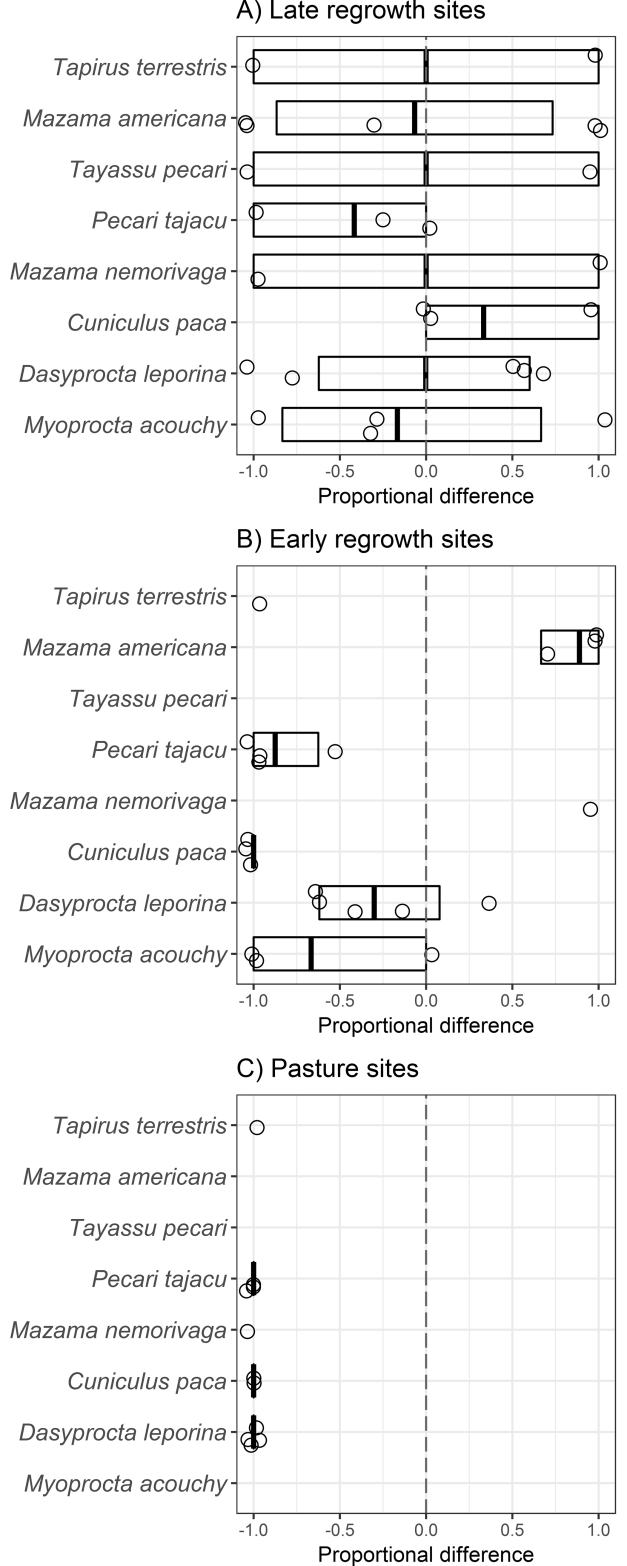
Differences along a lowland Amazon regrowth gradient for eight mammal species. Differences in detections from camera-trap videos taken in three regrowth classes (A to C, N = 5 sites each) compared with paired control sites (N = 5 sites each). Values ranging from less to more detections in regrowth sites, i.e. -1 (only present in control sites) to +1 (only present in regrowth sites). Boxplots show means and 95% confidence limits from the regrowth-control pairs estimated via nonparametric bootstrap.

The correlation between the species differences and regrowth class (control vs. late second-regrowth forest and control vs. early second-regrowth forest) was weak (Pearson, r = 0.23; P = 0.58), indicating that patterns in species composition differed between the regrowth stages (Fig 4). Body mass was the best model and received the strongest support for late second-regrowth forest (*w*_i_ = 0.99) and early second-regrowth forest (*w*_i_ = 0.98) (Table 4). In both early and late second-regrowth forest classes, the association between body mass and record change were negative (Table 4).

**Table 4:** Results of Akaike information criterion (AIC)-based model selection assessing the association between one measure of species sensitivity and a set of candidate GLMs. For each model, the sample-size adjusted AIC (AICc), Akaike differences (ΔAIC), Akaike weights (wi) and Sign are presented.

Response	Model	AIC_c_	ΔAIC	*w*_*i*_	Sign
Control *vs*. Late second-regrowth forest	Body mass	25.9	0	0.96	-
	Trophic guild	32.1	6.2	0.04	-
	Body mass + Trophic guild	50.7	24.8	0	+/+
	Home range	54.8	28.9	0	+
	Body mass + Home range	109.2	83.3	0	-/+
Control *vs*. Early second-regrowth forest	Body mass	36.2	0	0.99	-
	Trophic guild	45.3	9.1	0.01	+
	Body mass + Trophic guild	61.1	24.9	0	-/+
	Home range	61.5	25.5	0	+
	Body mass + Home range	116.1	79.9	0	-/+

## Discussion

Our results indicate that more intensely disturbed sites, such as abandoned pastures, and early (1–5 year) second-regrowth forests experience reduced richness and functional diversity of medium and large bodied terrestrial mammal species that are potentially important for the maintenance and regeneration of Amazon forests. Here we discuss our findings firstly in light of the sampling design adopted and then in relation to the potential for the regeneration of degraded Amazon forests.

### Sampling effort and mammal community

The difference between the observed and extrapolated species richness values obtained indicates that our sampling effort was sufficient to record all eight species in both control and regrowth sites. This finding was to be expected, as all eight species are widespread and ubiquitous across lowland Amazon forests [31, 41, 42, 45]. However, fewer species were detected in the more intensely disturbed sites (early secondary-regrowth and pasture). We believe that the absence of some species is not related to the sampling effort, but factors associated with anthropogenic disturbances such as hunting around the small holder properties [13, 21, 31].

Differences in sample effort are known to determine the number of species detected [54]. None of the target species were recorded in pasture sites, which also had a lower sampling effort (77 camera-trap days). This reduced sampling effort could contribute to the reduced number of species detected in pasture sites. Previous studies have shown that several of our study species do use pasture areas [60, 61] and we cannot exclude the possibility that additional effort would result in the detection of at least some species that may intermittently traverse pasture sites [40]. However, with the same effort as pasture sites (77 camera-trap days) we detected 38% and 75% of species in early and late second-regrowth sites (3 and 6 species respectively), which is still well above the zero detections in pasture sites. Given the stark reduction in pasture sites and our overall survey effort, we believe that the observed patterns in richness and functional diversity are representative of the true patterns (e.g. relative species rarity) in the different regrowth classes.

### Richness and functional diversity

We expected richness and functional diversity to decrease with the increasing land use intensity across the different regrowth stages [62]. As expected we found drastic reductions in richness and functional diversity in pasture areas, but considerably more variation in early and late second-regrowth areas. In a global review Bowen, McAlpine (7) showed that richness/abundances are variable in abandoned pasture, regrowth forest and control areas for diverse species groups (invertebrates, reptiles, birds and mammals). Our eight study species are found across different forest habitats throughout tropical and sub-tropical regions of South America [41, 63]. The eight species are therefore not strictly depended on the quality of forest habitat compared with other more specialist groups. As the species are found across a variety of habitats, a direct effect of differences in habitat quality in the different regrowth stages is therefore unlikely. Rather, our results support previous findings that show variation in community and species responses reflect differences in both species traits and human impacts [13, 31, 45].

We found that taxonomic and functional diversity was divergent among the regrowth areas. For example, the number of species was greater in late second-regrowth compared with early second-regrowth sites, but the functional diversity values were more similar. Although species were lost, the functional diversity of the mammal assemblage was retained at intermediate regrowth stages (early second-regrowth sites). The persistence of functional diversity in this group of mammals is good news for forest landscape restoration initiatives [5, 13].

Our findings support previous studies that show how the proportion of old-growth species increases with the age of secondary/regrowth forests [64, 65]. Indeed, our study area is representative of the best case scenario for species conservation/restoration as highlighted by Chazdon, Peres (64) “where the ratio of secondary to old-growth forest area is relatively low, older secondary forests have persisted, anthropogenic disturbance after abandonment is relatively low, seed-dispersing fauna are present, and old-growth forests are close to abandoned sites”. We found that the functional diversity of 8 key species returned to reference levels relatively quickly (after 5 years) in the early secondary regrowth areas. This rate of recovery is similar to that found in the absence of ongoing human disturbance in lowland forests of the Peruvian Amazon [65]. Although low level human disturbances continue in our study sites, our findings suggest that the proximity to vast areas of relatively intact forest enables the rapid return of the species when anthropogenic disturbance after abandonment is relatively low. This rapid recovery of functional diversity can enable the development of effective restoration actions within Amazon forest regrowth sites [5, 9, 13].

There are a number of options for forest restoration in regions where human populations are below 10/km^2^ [66]. The proximity to large areas of remnant forests and potential for agroforestry generate opportunities for both the restoration of ecosystem services and socio-economic development [5]. Different ecological, biophysical and socioeconomic factors correlate with the success of natural regeneration of tropical forests [67]. Within our study area there is a potential to generate US$ 2,958,800 a year from açai (*Euterpe oleracea*) agroforestry production close (<2 km) to rivers [68]. Although passive/natural regeneration is not without costs for local small-holders [69], the potential to integrate lucrative açai agroforestry and natural regeneration facilitated by functionally diverse terrestrial mammal assemblages in regrowth areas must not be overlooked.

### Trait correlates of degradation gradient sensitivity

We found that body mass was lost from the species assemblage along a degradation gradient. This supports previous findings demonstrating the loss (mainly due to hunting) of larger bodied seed dispersers from both regrowth areas and relatively pristine forest sites [8, 13, 23, 31]. As expected we found a loss of larger bodied species from Early second-regrowth sites, with only one species (*M*. *americana*) detected more frequently in these sites. This confirms our prediction of a loss of functional traits in more intensely regrowth areas.

In addition to the loss of functional traits from regrowth sites we also found a more surprising loss from the paired control sites. Although there are a number of non-mutually exclusive explanations for these losses from the forests bordering pasture and early regrowth sites, we propose edge effects as the most plausible explanation for the observed differences. The small bodied scatter-hoarding rodent *D*. *leporina* was the only species to be detected more frequently at the controls paired with pasture sites. All other species were either absent (*T*. *pecari*, *M*. *americana* and *M*. *agouchy*) or detected less frequently (*T*. *terrestris*, *P*. *tajacu*, *C*. *paca* and *M*. *nemorivaga*). More than 20 years of research shows that myriad edge effects permeate up to 150 m into Amazon forests [70]. Therefore the natural regeneration and/or restoration of regrowth habitats in Amazon small-holder properties (typically < 100 ha) that are largely covered by such distances will depend strongly on the ecological responses of species to habitat edges [71, 72].

Our study species show diverse responses to forest-non forest habitat edges in fragmented landscapes [22, 40]. In our study area the forest-degradation edges range from soft edges in the late secondary regrowth sites to hard and abrupt edges in the pasture sites. The loss of taxonomic and functional diversity, including body mass along this gradient is a challenge for the regeneration of regrowth areas. We found that the majority of the important terrestrial mammal seed dispersers avoid both pasture and paired control sites. Not only does pasture inhibit the establishment of seeds but the loss of dispersers mean there is a simultaneous reduction in seed entry [73]. Our findings support previous studies that show economic investment within an active management scenario are required to break regeneration barriers and facilitate natural regeneration around pasture areas [69, 71, 73, 74].

### Conclusions

Our findings generate new insight not only for understanding species ecology but also the potential role for this group in the regeneration of regrowth forest areas across the Amazon basin. Although we found a loss in richness and functional diversity with increasing disturbance intensity we also found that diversity in regrowth sites could be similar to control sites even in some early second-regrowth areas. These findings suggest that the functional diversity of these species (including the potential to act as regenerators of regrowth areas) can be retained in regrowth forest areas close to human small-holdings if there is both a low human population density and large areas of contiguous protected areas.

## Supporting information

S1 Text Study sites. (DOCX)

S1 Fig Study area rainfall. (DOCX)

S2 Fig Model semi-variograms. (DOCX)

S3 Fig Species accumulation curves from all, control and regrowth sites. (DOCX)

S4 Fig Species accumulation curves from early and late regrowth sites. (DOCX)

S1 Table Explanatory variables. (DOCX)
