# Supplementary material for: Diversity of terrestrial mammal seed dispersers along a lowland Amazon forest regrowth gradient
Source: PLoS One. 2018 Mar 16;13(3):e0193752. doi: 10.1371/journal.pone.0193752 (PMC5856264; doi:10.1371/journal.pone.0193752)
Supplement: S1 Text — (DOCX) [file pone.0193752.s001.docx]

S1 Text: Study sites.

Our study took place in 15 areas of regrowth on small holder properties in the center of the State of Amapá (Fig S1). The State of Amapá has the lowest deforestation rate in Brazil and > 70% of the Amapá receives some form of legal protection. Our study took place in small holder properties along rivers in the Araguari River Basin (Fig S1). There are some 54 small holder properties distributed along 180 km of river upstream of the nearest town (Porto Grande). There are no large scale agricultural developments or monocultures along the waterways and properties retain typically small (< 100 ha) areas of open land , which is cleared for small scale family agriculture, which focuses on açai, small scale production of fruits and vegetables for sustenance and limited commercial sale of regional produce (e.g. manioc flour) in local markets. There has never been any expansive clearcutting in the region and there are no monocultures (e.g. soy) or cattle production. All sites were at least 26 km from the nearest town, and all sites are surrounded by matrix of continuous closed canopy forest cover (Table S1). Pesticides and/or herbicides had never been used at any of the sites.


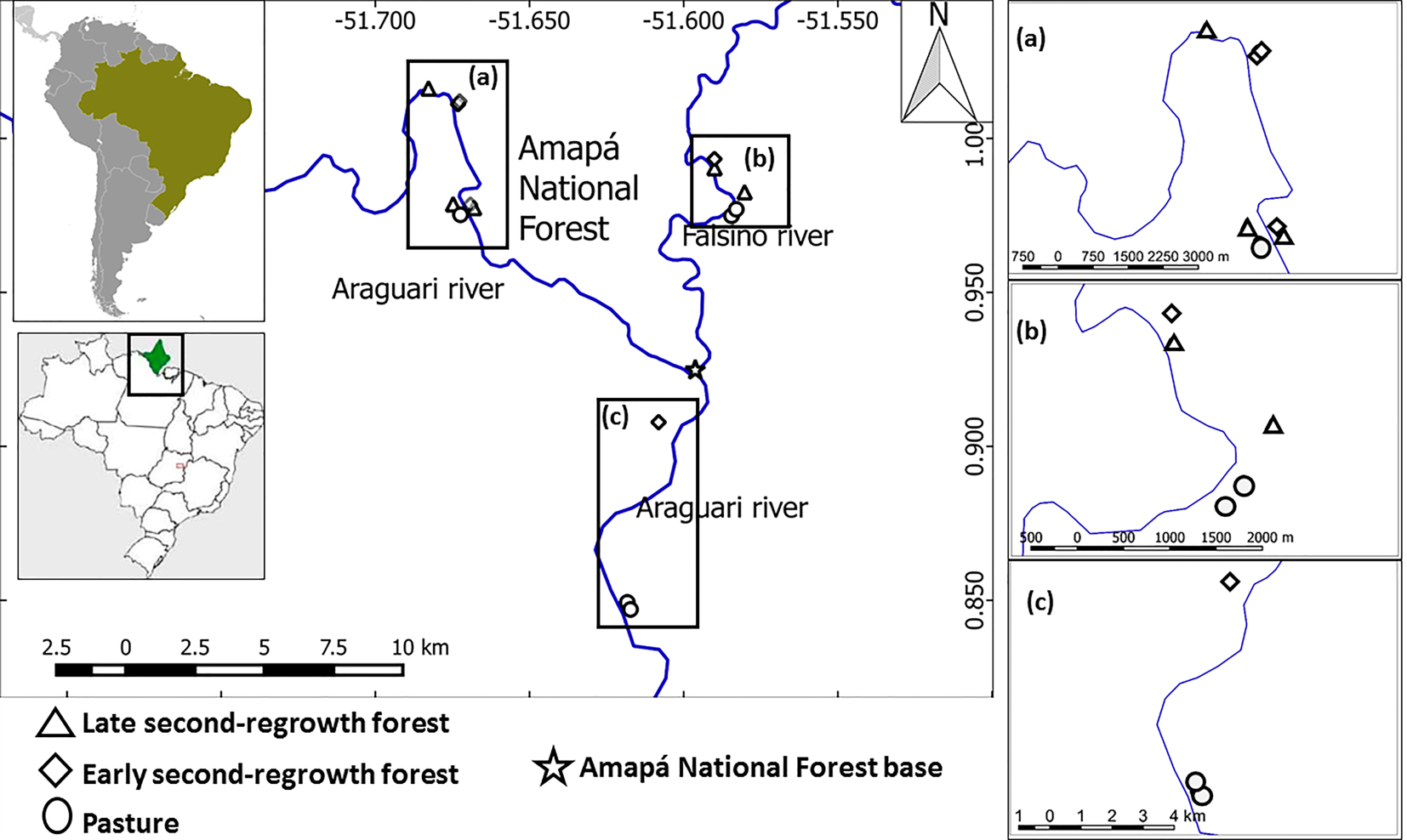


**
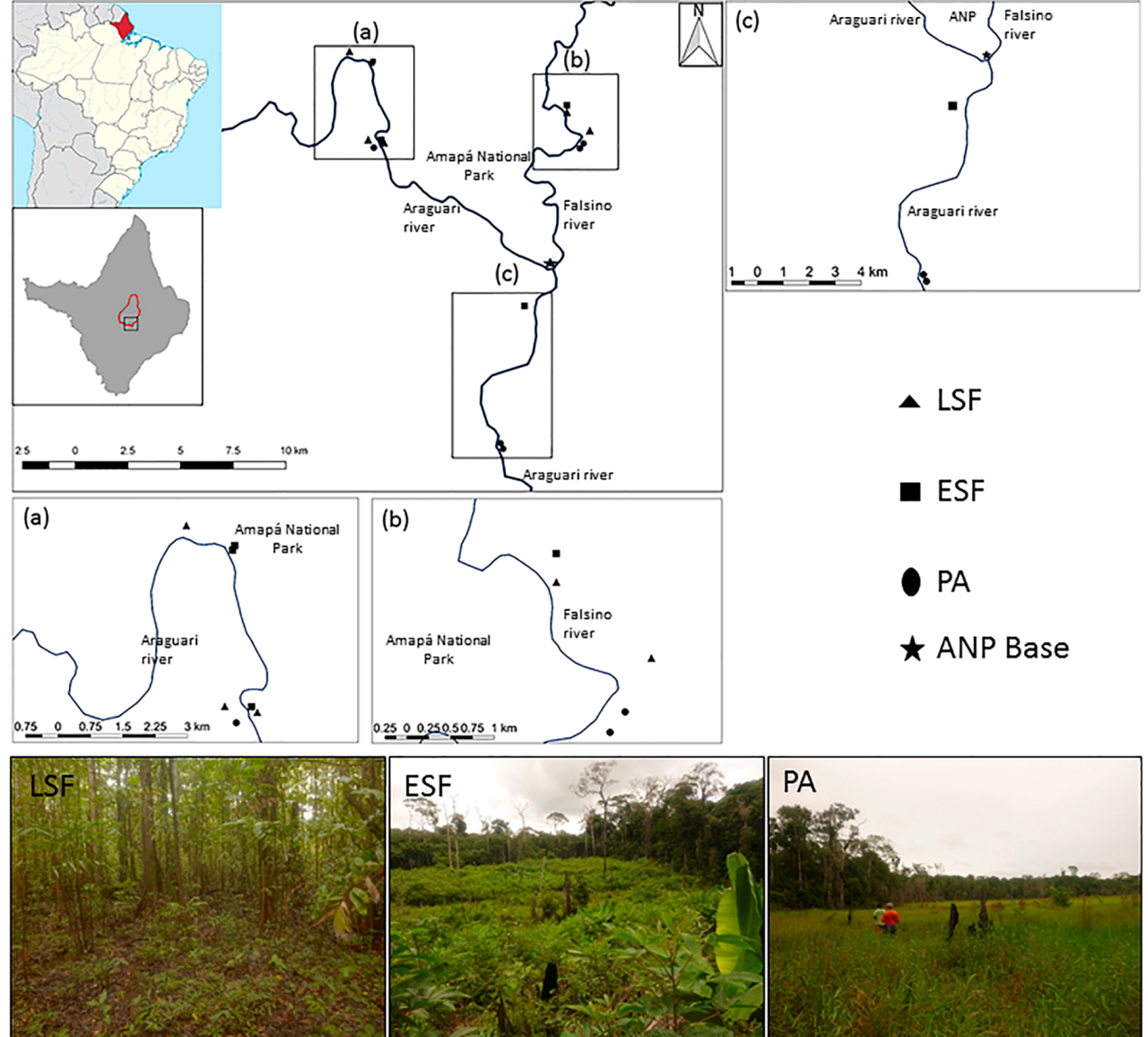
**

**Fig. S1.**  Map of the study area in the eastern Amazon. Showing the location of 15 study sites, grouped into three regrowth stages in the small holder properties close to rivers (solid blue lines): late second-regrowth forest (LSF, triangles), early second-regrowth forest (ESF, squares) and pasture (PA, circles).

Table S1. Forest regrowth site characteristics. Showing means with ranges in parentheses of values from the five camera-trap sites in each regrowth class.

| Regrowth class | Size (ha) | Distance to river (m) | % forest cover in a 1 km circular buffer | % forest cover in a 5 km circular buffer | Distance to nearest town (km) |
| --- | --- | --- | --- | --- | --- |
| Late regrowth | 5.8 (2.0 – 12.0) | 233.6 (150 - 460) | 91.2 (86.0 – 96.0) | 97.4 (96.4 – 98.5) | 39.5 (35.0 – 45.0) |
| Early regrowth | 2.4 (1.0 – 4.5) | 326.8 (150 – 500) | 90.6 (87.6 – 95.8) | 97.2 (96.4 – 98.4) | 38.5 (30.0 – 43.9) |
| Pasture | 8.7 (6.8 – 9.9) | 221.0 (170 – 315) | 88.5 (85.7 – 90.9) | 96.9 (95.5 – 98.4) | 32.8 (26.8 – 40.8) |
