# Supplementary material for: Diversity of terrestrial mammal seed dispersers along a lowland Amazon forest regrowth gradient
Source: PLoS One. 2018 Mar 16;13(3):e0193752. doi: 10.1371/journal.pone.0193752 (PMC5856264; doi:10.1371/journal.pone.0193752)
Supplement: S1 Fig — (DOCX) [file pone.0193752.s002.docx]

S1 Fig: Study area rainfall

Camera traps were installed and operational across the wet-dry transition (May – September 2016). During the survey period the total monthly rainfall was 168, 152, 171, 100 and 80 mm (monthly totals for May, June, July, August and September respectively, Fig. S2).

**
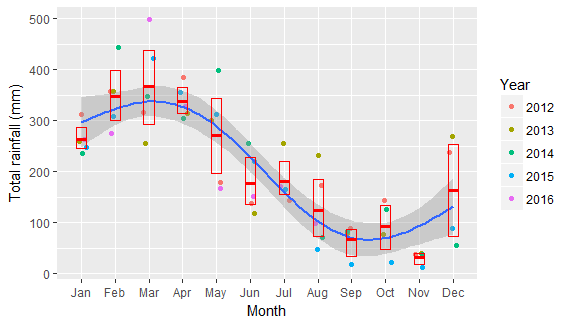
**

Figure S2. Monthly rainfall recorded close (36 km) to the Amapá National Forest study site. Weather station data available from the Brazilian National Water Agency (station ID: 8052000). Monthly totals are presented from five years (2012, 2013, 2014, 2015 and 2016). Boxplots show means and 95% confidence limits estimated via nonparametric bootstrap. The blue line and shaded areas are the mean value and 95% confidence intervals from a GAM model illustrating the trend in rainfall.

ANA. Sistema de Monitoramento Hidrológico (Hydrological Monitoring System). Agência Nacional de Águas[[nl]]National Water Agency, Available at <http://hidroweb.ana.gov.br> 2016 [08.01.2017].
