# Supplementary material for: Diversity of terrestrial mammal seed dispersers along a lowland Amazon forest regrowth gradient
Source: PLoS One. 2018 Mar 16;13(3):e0193752. doi: 10.1371/journal.pone.0193752 (PMC5856264; doi:10.1371/journal.pone.0193752)
Supplement: S2 Fig — (DOCX) [file pone.0193752.s003.docx]

S2 Fig: Model Semivariograms

Here we use semi-variograms to test for any residual spatial dependence in the different analyses presented in the main manuscript.

Uncorrelated residuals should give a more or less flat semi-variogram, while unmodelled spatial auto-correlation (spatial dependence) usually results in a semi-variogram which increases sharply before eventually plateauing [1, 2]. In the current case, the semi-variograms suggest no spatial dependence in the models presented in the main text.

S2 Semi-variograms from (a) linear regressions and (b) ANOVAs presented in the main text. Sample semi-variograms and simulation envelopes under random permutation of model residuals. Distances calculated from geographic coordinates (decimal degrees, 0.01 ≈ 900m).

a)

| Sample | Response | |
| --- | --- | --- |
|  | Species richness | Functional dispersion |
| 30 sites (control and degraded) | 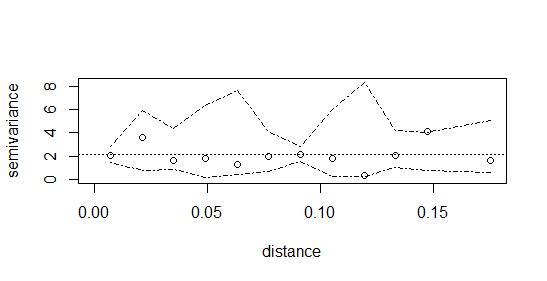 | 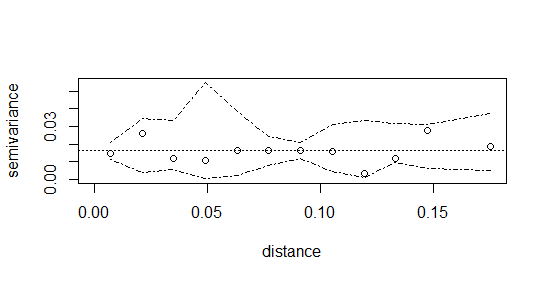 |
| 15 Degraded sites | 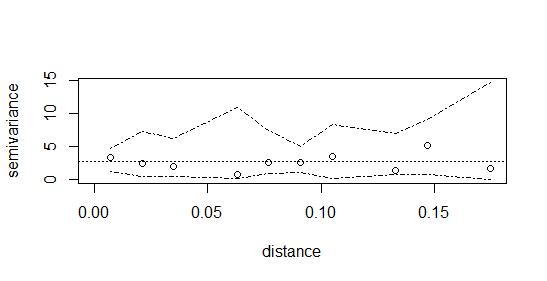 | 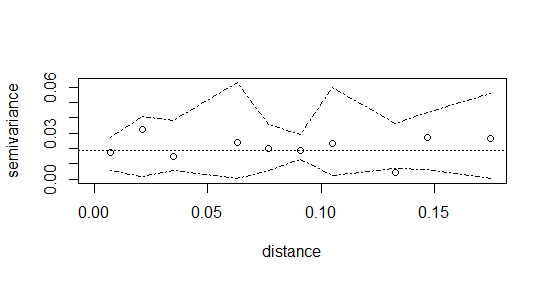 |

b)

| Response | Semi-variogram of model residuals |
| --- | --- |
| Species richness | 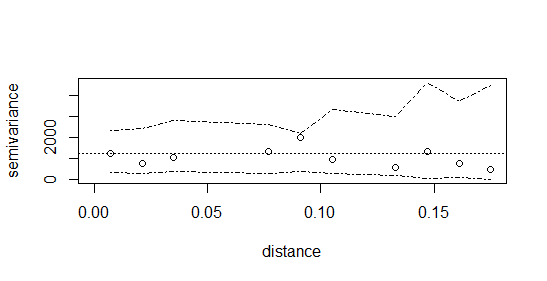 |
| Functional dispersion | 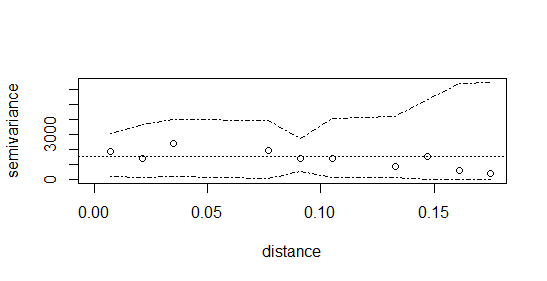 |

Table S4.2 Semi-variograms from information-theoretic analysis presented in the main text. Sample semi-variograms and simulation envelopes under random permutation of model residuals. Distances calculated from geographic coordinates (decimal degrees, 0.01 ≈ 900m).

| Main Table | Model | | | | |
| --- | --- | --- | --- | --- | --- |
|  | Hunting | Forest cover | Land use | Global | Final |
| 2a | 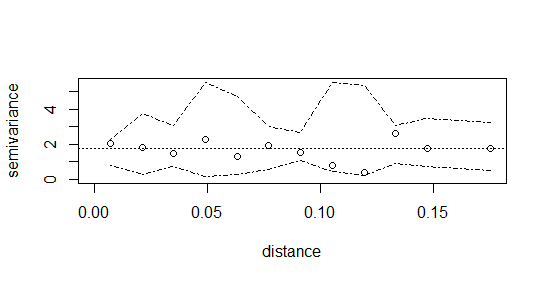 | 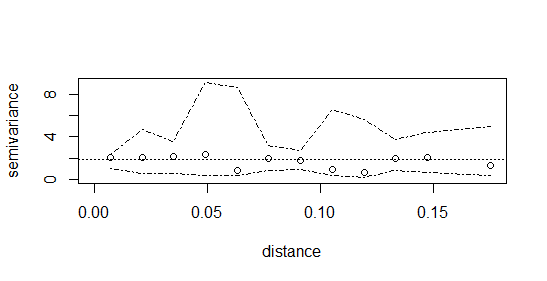 | 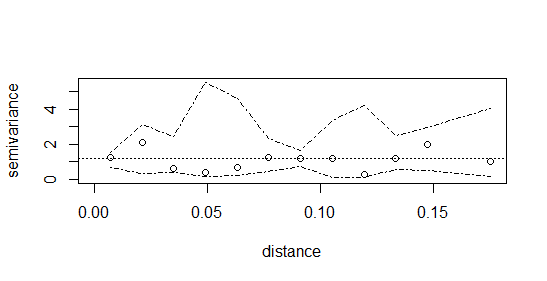 | 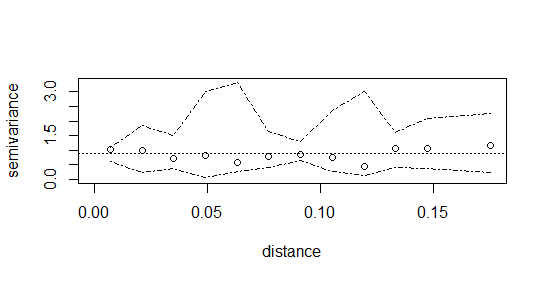 | 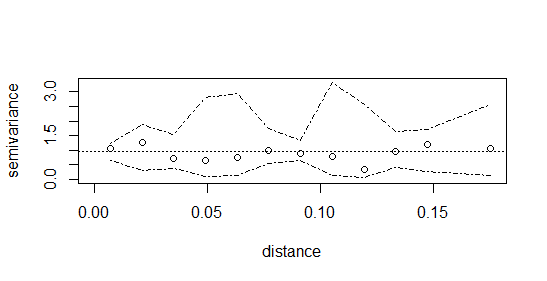 |
| 2b | 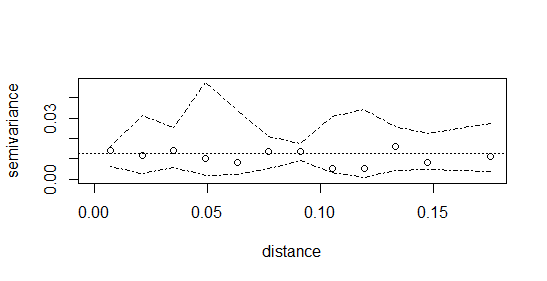 | 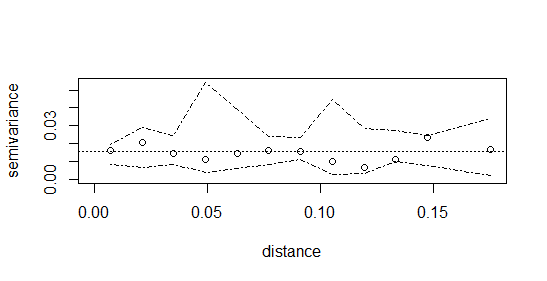 | 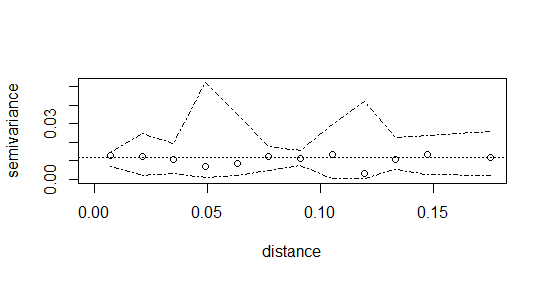 | 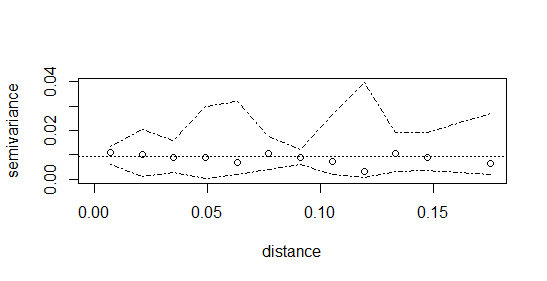 | 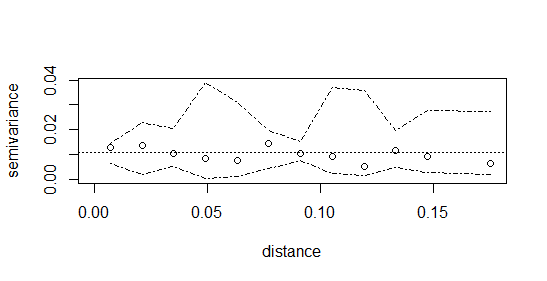 |
| 3a | 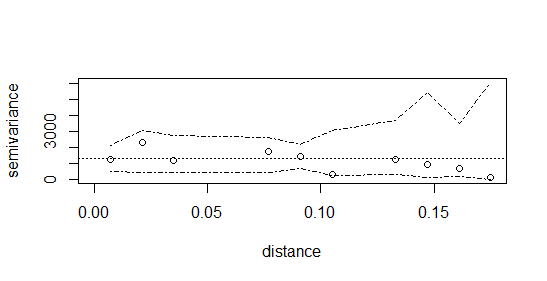 | 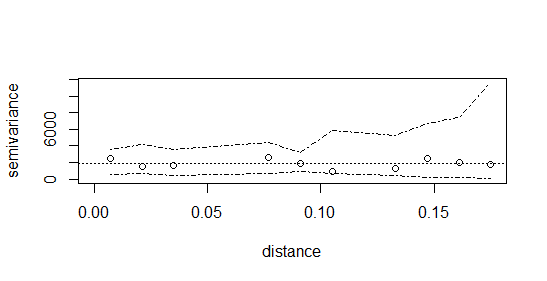 | 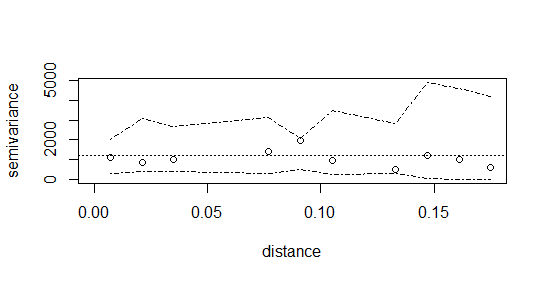 | 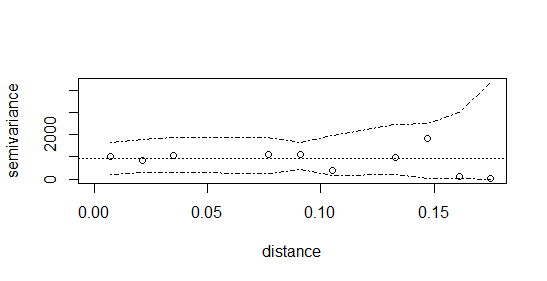 | 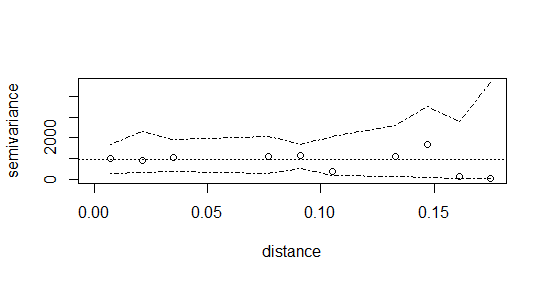 |
| 3b | 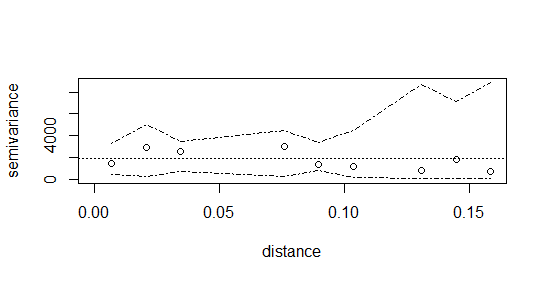 | 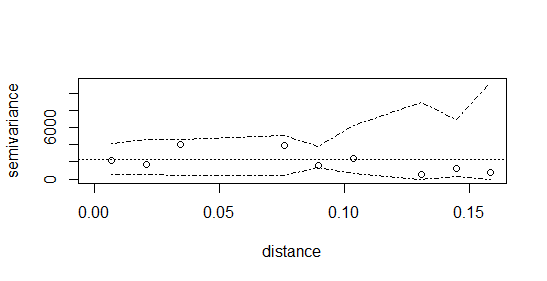 | 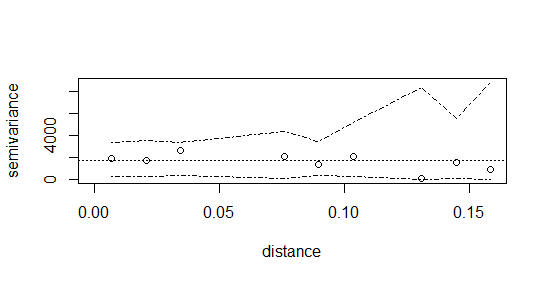 | 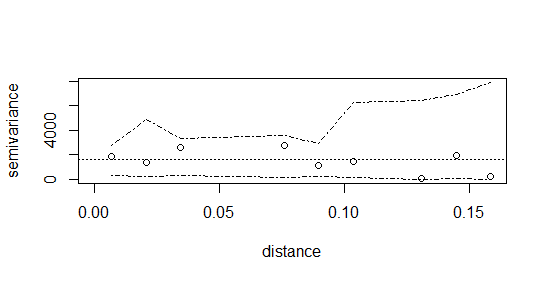 | 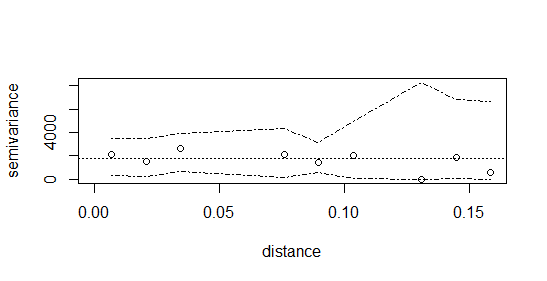 |

References

1. Dale MR, Fortin M-J. Spatial analysis: a guide for ecologists: Cambridge University Press; 2014.

2. Dormann CF. Effects of incorporating spatial autocorrelation into the analysis of species distribution data. Global ecology and biogeography. 2007;16(2):129-38.
