# Supplementary material for: Diversity of terrestrial mammal seed dispersers along a lowland Amazon forest regrowth gradient
Source: PLoS One. 2018 Mar 16;13(3):e0193752. doi: 10.1371/journal.pone.0193752 (PMC5856264; doi:10.1371/journal.pone.0193752)
Supplement: S4 Fig — (DOCX) [file pone.0193752.s005.docx]

S4 Fig: Species accumulation curves from early and late regrowth sites.


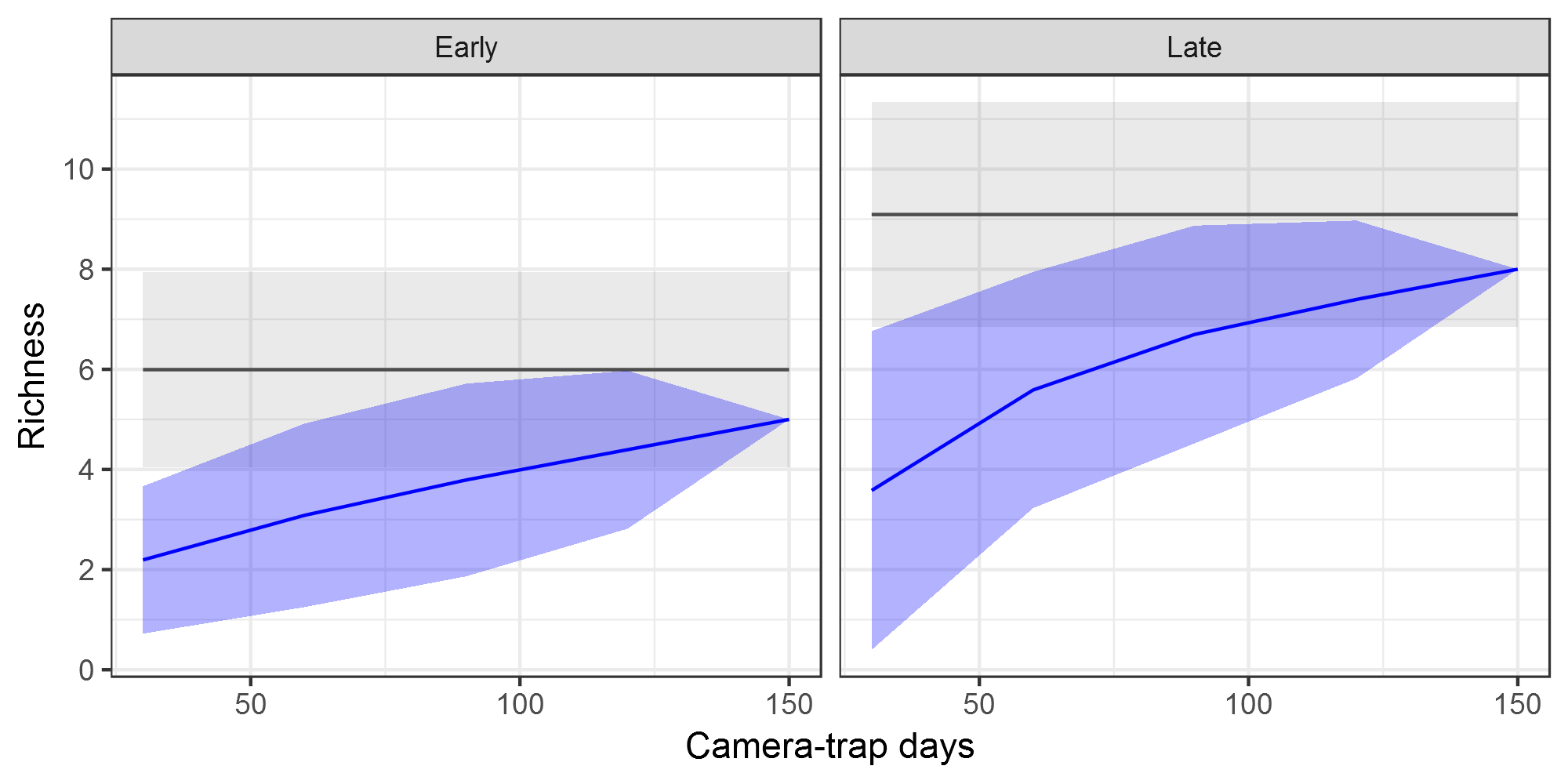


Figure S4: Species accumulation curve. Detection of species recorded with camera-traps randomized 1000 times and results used to derive mean (solid blue line) and 95% confidence intervals (light blue polygon). Bootstrap estimates of extrapolated species richness and 95% confidence intervals are shown with black line and light gray shaded area, respectively. Comparison between early (n = 5) and late (n = 5) secondary-regrowth sites.
