# Supplementary material for: Diversity of terrestrial mammal seed dispersers along a lowland Amazon forest regrowth gradient
Source: PLoS One. 2018 Mar 16;13(3):e0193752. doi: 10.1371/journal.pone.0193752 (PMC5856264; doi:10.1371/journal.pone.0193752)
Supplement: S1 Table — (DOCX) [file pone.0193752.s006.docx]

S1 Table: Explanatory variables

| Hypothesis | Model variables | Source | Description/Ecological relevance | Supporting references |
| --- | --- | --- | --- | --- |
| Hunting pressure | Distance to river | In situ GPS | Continuous. Euclidian distance from camera location to river bank. Access to sites via rivers, therefore sites closer to rivers expected to have higher pressure. | (Peres and Lake 2003; Peres and Terborgh 1995) |
|  | Distance to town | In situ GPS | Continuous. Distance along river from site to local town. Local town is a likely source of additional anthropogenic/hunting pressure. Sites closer to the town expected to have higher pressure. | (Michalski and Peres 2007; Peres and Lake 2003; Peres and Terborgh 1995) |
|  | Distance to house | In situ GPS | Continuous. Euclidian distance from house to camera location. Sites closer to houses expected to have higher pressure. | (Peres and Lake 2003; Peres and Terborgh 1995) |
|  | Presence | In situ interview | Categorical factor with 3 levels: abandoned, semi-present (e.g. weekends/holidays) or permanent. We expect species number and functional diversity to decrease with increasing permanence (absent to semi-permanent to permanent). | (Peres 2000; Peres and Lake 2003; Peres and Terborgh 1995) |
| Forest cover* | Cover 50m | (Hansen et al. 2013) | Continuous. Percentage forest cover within 50m of camera location. Higher cover percentages expected to have increased number of species and functional diversity. | (Michalski and Peres 2007; Prist et al. 2012) |
|  | Cover 1km | (Hansen et al. 2013) | Continuous. Percentage forest cover within 1km of camera location. Higher cover percentages expected to have increased number of species and functional diversity. | (Michalski and Peres 2007; Prist et al. 2012) |
|  | Cover 5km | (Hansen et al. 2013) | Continuous. Percentage forest cover within 5km of camera location. Higher cover percentages expected to have increased number of species and functional diversity. | (Michalski and Peres 2007; Prist et al. 2012) |
| Land use | Regrowth stage | In situ interview | Categorical factor with four levels. **Control sites** and three levels of regrowth class derived from the land-use history**: late second-regrowth forest** (N = 5, most recent human disturbance between 20 and 25 years), **early second-regrowth** (N = 5, most recent human disturbance between 1 and 5 years), and **pasture** (N = 5, recently cleared and abandoned pasture areas dominated by grasses/herbs but that had never been used to raise livestock, with the most recent disturbance between 1 and 17 years). | (Michalski and Peres 2007; Peres 1999) |
|  | Time since last use | In situ interview | Continuous. | (Michalski and Peres 2007; Prist et al. 2012) |

*Images of forest cover, forest loss and forest gain from 2000 to 2015 were downloaded from: <https://earthenginepartners.appspot.com/science-2013-global-forest> accessed 9 September 2017, details in Hansen et al. (2013). Forest cover for 2015 within the different distances was obtained using functions in the R package “gfcanalysis” (https://CRAN.R-project.org/package=gfcanalysis).

References

Hansen, M.C., Potapov, P.V., Moore, R., Hancher, M., Turubanova, S.A., Tyukavina, A., Thau, D., Stehman, S.V., Goetz, S.J., Loveland, T.R., Kommareddy, A., Egorov, A., Chini, L., Justice, C.O., Townshend, J.R.G., 2013. High-Resolution Global Maps of 21st-Century Forest Cover Change. Science 342, 850-853.

Michalski, F., Peres, C.A., 2007. Disturbance-mediated mammal persistence and abundance-area relationships in Amazonian forest fragments. Conservation Biology 21, 1626-1640.

Peres, C.A., 1999. Nonvolant mammal community structure in different Amazonian forest types, In Mammals of the Neotropics. eds J.F. Eisenberg, K.H. Redford, pp. 564-581. University of Chicago Press, Chigago.

Peres, C.A., 2000. Effects of subsistence hunting on vertebrate community structure in Amazonian forests. Conservation Biology 14, 240-253.

Peres, C.A., Lake, I.R., 2003. Extent of nontimber resource extraction in tropical forests: accessibility to game vertebrates by hunters in the Amazon basin. Conservation Biology 17, 521-535.

Peres, C.A., Terborgh, J.W., 1995. Amazonian nature reserves: an analysis of the defensibility status of existing conservation units and design criteria for the future. Conservation Biology 9, 34-46.

Prist, P.R., Michalski, F., Metzger, J.P., 2012. How deforestation pattern in the Amazon influences vertebrate richness and community composition. Landscape Ecology 27, 799-812.
